# Comparative genomic analysis and characterization of incompatibility group FIB plasmid encoded virulence factors of *Salmonella enterica* isolated from food sources

**DOI:** 10.1186/s12864-017-3954-5

**Published:** 2017-08-02

**Authors:** Bijay K. Khajanchi, Nur A. Hasan, Seon Young Choi, Jing Han, Shaohua Zhao, Rita R. Colwell, Carl E. Cerniglia, Steven L. Foley

**Affiliations:** 1 U.S. Food and Drug Administration, National Center for Toxicological Research, Jefferson, AR USA; 2 Center of Bioinformatics and Computational Biology, University of Maryland Institute of Advanced Computer Studies, University of Maryland, College Park, MD USA; 3 CosmosID, Inc., Rockville, MD USA; 4 U.S. Food and Drug Administration, Center for Veterinary Medicine, Laurel, MD USA

**Keywords:** *Salmonella enterica*, Incompatibility group FIB plasmid, Iron acquisition systems (SitABCD and aerobactin), Human intestinal epithelial (Caco-2) cells, Virulence, Single nucleotide polymorphism (SNP), Whole genome sequencing (WGS)

## Abstract

**Background:**

The degree to which the chromosomal mediated iron acquisition system contributes to virulence of many bacterial pathogens is well defined. However, the functional roles of plasmid encoded iron acquisition systems, specifically Sit and aerobactin, have yet to be determined for *Salmonella* spp. In a recent study, *Salmonella enterica* strains isolated from different food sources were sequenced on the Illumina MiSeq platform and found to harbor the incompatibility group (Inc) FIB plasmid. In this study, we examined sequence diversity and the contribution of factors encoded on the IncFIB plasmid to the virulence of *S*. *enterica*.

**Results:**

Whole genome sequences of seven *S*. *enterica* isolates were compared to genomes of serovars of *S*. *enterica* isolated from food, animal, and human sources. SeqSero analysis predicted that six strains were serovar Typhimurium and one was Heidelberg. Among the *S.* Typhimurium strains, single nucleotide polymorphism (SNP)-based phylogenetic analyses revealed that five of the isolates clustered as a single monophyletic *S*. Typhimurium subclade, while one of the other strains branched with *S*. Typhimurium from a bovine source. DNA sequence based phylogenetic diversity analyses showed that the IncFIB plasmid-encoded Sit and aerobactin iron acquisition systems are conserved among bacterial species including *S. enterica*. The IncFIB plasmid was transferred to an IncFIB plasmid deficient strain of *S*. *enterica* by conjugation. The transconjugant SE819::IncFIB persisted in human intestinal epithelial (Caco-2) cells at a higher rate than the recipient SE819. Genes of the Sit and aerobactin operons in the IncFIB plasmid were differentially expressed in iron-rich and iron-depleted growth media.

**Conclusions:**

Minimal sequence diversity was detected in the Sit and aerobactin operons in the IncFIB plasmids present among different bacterial species, including foodborne *Salmonella* strains. IncFIB plasmid encoded factors play a role during infection under low-iron conditions in host cells.

**Electronic supplementary material:**

The online version of this article (doi:10.1186/s12864-017-3954-5) contains supplementary material, which is available to authorized users.

## Background

Salmonellosis, the leading bacterial foodborne illness in the United States, is associated with consumption of food contaminated with *Salmonella* spp. [1]. In the US alone, it is estimated that more than 1 million *Salmonella* infections occur annually, resulting in approximately 20,000 hospitalizations and 400 deaths [1] . *S. enterica* has been identified as the source of multiple outbreaks associated with meat and poultry products in the US and other countries [2]. Approximately 42% of reported cases occur in children under 10 years of age [3]. The most common manifestation of *Salmonella* infection is gastroenteritis. However, in severe cases, septicemia or organ system infections can develop [4]. Some *S*. *enterica* serovars cause more invasive infections than others; for example, a previous study demonstrated serovars Heidelberg and Typhimurium were more invasive, i.e., representing 13% and 6% of infections, respectively [5]. Thus, it is important to understand those factors contributing to the more severe manifestations of this disease.

Incompatibility group (Inc) FIB plasmids (also commonly known as ColV plasmids) can encode both virulence factors and antimicrobial resistance genes [6, 7]. These plasmids have been shown to contribute to the virulence of extra-intestinal pathogenic *Escherichia coli* (ExPEC) [7–9]. It has been demonstrated that horizontal gene transfer of IncFIB plasmid resulted in the emergence of a dominant avian clonal type of *S*. *enterica*, namely serovar Kentucky [10]. In the same study, investigators examined distribution of these plasmids among 902 *Salmonella* isolates from different poultry sources. The IncFIB plasmid was found to occur predominantly in serovar Kentucky (72.9% of isolates tested), followed by Typhimurium (15%) and Heidelberg (1.7%); the latter two serovars are among the most commonly associated with disease in humans [10]. Results of the study demonstrated that acquisition of the IncFIB plasmid by *S*. *enterica* serovar Kentucky significantly increased its ability to colonize the chicken cecum and cause extra-intestinal disease [10]. The potential for horizontal gene transfer of virulence and fitness related factors encoded by the IncFIB plasmid between *Salmonella* and other enteric bacteria exists in isolates recovered from food sources. This is a potential concern for human health as these virulent species of bacteria can be acquired by humans via contaminated foods.

A previous study carried out in our laboratory showed that IncFIB plasmids from *Salmonella* carry genes associated with virulence, including iron acquisition transport systems, such as the Fur (ferric uptake regulator), regulated iron ABC transporter (*sitABCD*) and the aerobactin iron acquisition (*iucABCD-iutA*) system [6]. Fur, a transcriptional and post-transcriptional regulator that senses iron in the environment, plays a crucial role in the colonization and pathogenicity in many bacteria [11–13]. The role of the chromosomal mediated Sit iron acquisition system in virulence of many bacterial pathogens is well defined [14, 15]. However, functional roles of iron acquisition transport systems encoded by the IncFIB plasmid have yet to be determined for *Salmonella*. The main objectives of this study were: i) to analyze and compare genomes of IncFIB plasmid containing *S*. *enterica* strains isolated from turkeys and chicken; ii) to determine the sequence diversity of IncFIB plasmid encoded Sit and aerobactin operons in different bacteria including *Salmonella*; iii) to evaluate the degree of contribution of IncFIB plasmid encoded factors in virulence of *S*. *enterica*.

Iron is an essential cofactor for various metabolic enzymes associated with important biological pathways in both microorganisms and host [16]. For example, iron influences butyrate production by modulating expression of butyryl-coenzyme A (CoA):acetate CoA-transferase enzyme of butyrate producer such as *Roseburia intestinalis* [17]. In the recent past, butyrate has been identified as an important metabolite for maintaining healthy microbiota in human gut [17]. Iron is also an important growth factor for pathogenic bacteria, with iron concentrations of 10^−6^ to 10^−7^ M required by most microorganisms to carry out metabolic processes, including electron transport, glycolysis, DNA synthesis, and defense against toxic reactive oxygen intermediates (ROI) [18]. In response to infection, eukaryotic hosts utilize strategies to limit iron availability to pathogens. Specific host proteins, such as transferrin, lactoferrin, and ferritin, generally bind to haem to form a complex within haemoprotein, resulting in unavailability of free iron [19, 20]. Additionally, iron is required by macrophages and/or other host cells for the production of ROI and reactive nitrogen intermediate (RNI) which, both aid in the elimination of pathogens [16]. On the other hand, pathogens have evolved to encode various iron acquisition systems in the chromosome and plasmids, including those mentioned above, to sequester iron from the host to establish an infection [21, 22]. For instances, it has shown that aerobactin defective mutants of avian pathogenic *E. coli* (APEC) and uropathogenic *E. coli* (UPEC) showed reduced virulence in a chicken infection model [9]. Previous study demonstrated that SitABCD iron acquisition system encoded by chromosome of *S*. Typhimurium required for complete virulence of this pathogen [15]. Plasmid encoded Sit and aerobactin iron acquisition systems homologs have been identified in *Cronobacter* spp. which possesses several virulence associated genes [23, 24].

Recently, we sequenced seven *S. enterica* isolates from different food sources such as turkey and chicken, six of which contained IncFIB plasmids [25]. In the present study, we used single nucleotide polymorphism (SNP)-based phylogenetic analysis to compare genome sequences of these isolates to selected *S*. *enterica* serovars isolated from food, animal, and human sources available on the public data base. Additionally, we conducted phylogenetic analysis of the IncFIB plasmid encoded Sit and aerobactin operons to determine sequence diversity of these factors. The expression of genes within these operons was also evaluated at the transcriptional level following growth in iron-rich or iron-depleted media. To delineate functional roles of IncFIB plasmid encoded factors in virulence, invasion, and persistence of *S*. *enterica*, human intestinal epithelial cells (Caco-2) were infected with IncFIB negative recipient SE819, transconjugant SE819::IncFIB, and wild type (plasmid donor) SE163A strains. The results provide data useful in understanding the functional roles of iron acquisition systems encoded by IncFIB plasmids of *S. enterica* and related pathogens.

## Methods

### Bacterial strains and growth medium

Bacterial isolates included in this study (Additional file 1: Table S1) were sub-cultured on sheep’s blood agar plates (Remel, Lenexa, KS). Single colonies were picked and grown overnight in Luria-Bertani (LB) medium supplemented with the following antibiotics: tetracycline (32 μg/mL); kanamycin (32 μg/mL); and nalidixic acid (64 μg/mL). Sodium azide (350 μg/mL) (Sigma-Aldrich, St. Louis, MO) was used in the media for conjugation. Ferric chloride (100 μM) and 2,2^/^−bipyridyl (200 μM) (Sigma-Aldrich) were added to LB broth to prepare iron-rich and iron-depleted growth media, respectively.

### Whole genome sequencing (WGS)

Genomic DNA was extracted with the DNeasy Blood and Tissue Kit (Qiagen, Valencia, CA), and sequenced at the University of Arkansas for Medical Sciences DNA Sequencing Core Facility (Little Rock, AR). To construct DNA libraries, the Nextera XT DNA Sample Prep Kit was employed, following manufacturer’s instructions (Illumina, San Diego, CA). Sequencing was performed on the Illumina MiSeq with 2 × 250 paired-end reads [25]. CLC Genomics Workbench (version 8.5.1; Qiagen, Germantown, MD) was used for trimming and de novo assembly of paired-end reads. Contigs less than 200 nucleotides were excluded from analysis. Draft genomes of *S*. *enterica* were annotated with the Rapid Annotation using Subsystem Technology (RAST) [26], Pathosystems Resource Integration Center (PATRIC) [27], and NCBI Prokaryotic Pipeline (PGAP). More detailed descriptions of the sequencing experiments were reported by Khajanchi et al. (2016) [25]. The *S*. *enterica* WGS data were submitted to NCBI and the accession numbers were listed in Table 1. Serovars of the seven *S*. *enterica* isolates were predicted using SeqSero, a WGS based tool (www.denglab.info/SeqSero) [28].

**Table 1:** *Salmonella enterica* isolates employed in the SNP analysis of this study

Strain	Serovar	Isolation Country	State	Source	Isolation Date	Accession
SE163A^c^	Typhimurium^b^	USA	OH	Turkey diagnostic	2002	LSZD00000000
SE696A^c^	Typhimurium^b^	USA	MW	Turkey processing	2000	LXHA00000000
SE710A^c^	Typhimurium^b^	USA	ND	Turkey diagnostic	1992	LXGZ00000000
SE819^c^	Heidelberg^b^	USA	MD	Turkey	2002	LSZE00000000
SE397^c^	Typhimurium^b^	USA	INA^a^	Chicken carcass	1999	LYRR00000000
SE452^c^	Typhimurium^b^	USA	INA	Ground turkey	1999	LYRS00000000
SE478^c^	Typhimurium^b^	USA	INA	Turkey	1999	LYRT00000000
SESL476	Heidelberg	USA	MN	Food	2003	NC_011083.1
SECVM20752	Heidelberg	USA	NC	Turkey	2002	AMNR00000000
SE21381	Heidelberg	USA	CT	Food	2002	AMMZ00000000
SECVM24359	Heidelberg	USA	MO	Turkey	2003	AMNP00000000
SE29169	Heidelberg	USA	CT	Food	2003	APIX00000000
SE41563	Heidelberg	USA	OR	Human	2011	AJGX00000000a
SE41565	Heidelberg	USA	WA	Food	2011	AJHA00000000a
SE41567	Heidelberg	USA	WA	Food	2011	AMNG00000000
SE41573	Heidelberg	USA	OH	Human	2011	AJGY00000000a
SE41576	Heidelberg	USA	OH	Human	2011	AMNH00000000
SE82–2052	Heidelberg	USA	ME	Human	1982	AMMX00000000b
SEN1536	Heidelberg	USA	GA	Food	2004	AKYN00000000
SEN15757	Heidelberg	USA	GA	Food	2007	AMND00000000
SEN18393	Heidelberg	USA	CT	Food	2008	AMNF00000000
SEN19992	Heidelberg	USA	CA	Food	2009	AMMC00000000
SEN26457	Heidelberg	USA	CO	Food	2010	AMNE00000000
SEN29341	Heidelberg	USA	MN	Food	2011	AMNJ00000000
SEN4403	Heidelberg	USA	CA	Food	2005	AMMT00000000
SEN4496	Heidelberg	USA	CO	Food	2005	AMMQ00000000
SESARA31	Heidelberg	USA	MD	Swine	1987	AMLV00000000
SESARA32	Heidelberg	USA	TX	Dog	1986	AMLU00000000
SESARA37	Heidelberg	USA	CO	Turkey	1987	AMLR00000000
SEN13–01290	Heidelberg	Canada	Quebec	Food	2012	CP012930.1
SE12–4374	Heidelberg	Canada	Quebec	Human	2012	CP012924.1
SECFSAN002064	Heidelberg	USA	INA	Human	2012	CP005995.1
SECFSAN002069	Heidelberg	USA	WA	Chicken	2012	CP005390.2
SEB182	Heidelberg	France	INA	Cattle	INA	CP003416.1
SECVM_N41746	Heidelberg	USA	NY	Food	2012	NZ_JYYX01000033.1
SECVM_N42472	Heidelberg	USA	GA	Food	2012	NZ_JYZU01000053.1
SECVM_N37938	Heidelberg	USA	NY	Food	2011	NZ_JYWR01000049.1
SECVM_N30683	Heidelberg	USA	NM	Food	2011	NZ_JYUW01000029.1
SET000240	Typhimurium	Japan	INA	Human	2000	AP011957.1
SELT2	Typhimurium	INA	INA	Human	1940s	NC_003197.1
SECFSAN001921	Typhimurium	INA	INA	Food	INA	CP006048.1
SECDC_H2662	Typhimurium	INA	INA	Human	1997	CP014979.1
SEUSDA-ARS-USMARC-1808	Typhimurium	USA	INA	INA	INA	CP014969.1
SERM9437	Typhimurium	USA	CO	Food	2009	CP012985.1
SESA972816	Typhimurium	China	INA	Cattle	2002	CP007484.1
SECDC_2009K-1640	Typhimurium	USA	INA	Human	2009	CP014975.1
SECDC_2010K-1587	Typhimurium	USA	INA	Human	2006	CP014965.1
EUSDA-ARS-USMARC-1810	Typhimurium	USA	INA	Cattle	2005	CP014982.1
EUSDA-ARS-USMARC-1880	Typhimurium	USA	INA	Cattle	2003	CP014981.1
SECVM29188	Kentucky	USA	GA	Food	2003	CP001122.1
SECDC191	Kentucky	INA	INA	INA	INA	NZ_ABEI00000000.1
SESK222-32B	Kentucky	INA	INA	Food	2005	NZ_JUIU00000000.1

^a^INA; information not available

^b^ *Salmonella* serovars were predicted using SeqSero, a WGS-based tool [28]

^c^Seven *S*. *enterica* isolates (SE163A, SE696A, SE710A, SE452, SE478, SE397 and SE819) were sequenced in this study; the genome sequences of the rest of the 45 *Salmonella* isolates used for SNP analysis were obtained from a public data base

### Single nucleotide polymorphism (SNP) analysis

A phylogenetic tree of the sequenced *Salmonella* genomes (*n* = 52) was constructed with the parsnp program (Harvest software) [29], which identifies core genomes across isolates and builds a phylogeny using maximum likelihood and core single nucleotide polymorphisms (SNPs). Seven *S*. *enterica* isolates (SE163A, SE696A, SE710A, SE452, SE478, SE397 and SE819) were sequenced in our study; the genome sequences of the rest of the 45 *Salmonella enterica* strains isolated from food, animal, and human sources were obtained from a public data base. Detail information of the isolates used in the SNP analysis was listed in the Table 1.

### Phylogenetic analysis of iron acquisition systems

Composite phylogenetic trees were generated by averaging pair-comparisons of DNA sequences of the *S. enterica* and *E. coli* strains harboring IncFIB plasmids for each of the genes in either Sit (*sitABCD*) or aerobactin (*iucABCD-iutA*) operons, based on unweighted pair group means, employing the average (UPGMA) clustering algorithm, BioNumerics (version 7.6, Applied Maths, Kortrijk, Belgium).

### Conjugation and in vitro passage

Conjugation experiments were performed as described previously [30], with some modifications that were specifically employed for this study. To obtain a transconjugant that only contain IncFIB plasmid in SE819, we performed the conjugation experiments in two steps. In the first conjugation experiment, plasmids present in the wild type *S. enterica* SE163A were transferred to the sodium azide resistant recipient *E. coli* J53 using a plate mating strategy [31]. Briefly, the donor and recipient strains were cross streaked on selective LB agar plates containing sodium azide (350 μg/mL) and kanamycin (32 μg/mL) and incubated at 37 °C. After approximately 48 h, the cells from the intersection were collected and re-streaked onto selective plates containing sodium azide (350 μg/mL) and kanamycin (32 μg/mL). Individual colonies were picked and sub-cultured onto MacConkey agar. Subsequently, colorless colonies were picked form selective plate and transconjugants were confirmed by PCR targeting of the plasmid specific genes present in different plasmids in the donor SE163A strain (IncFIB, IncA/C and IncX4) [10, 32]. Transconjugants containing IncFIB but lacking the other plasmids were selected and stored at −80 °C. In the second conjugation experiment, a single colony of *E. coli* J53::IncFIB (Additional file 1: Table S1) and *S*. *enterica* SE819 lacking IncFIB plasmid were grown in LB medium overnight at 37 °C. The donor (*E. coli* J53::IncFIB) and recipient (SE819) strains were mixed at a ratio of 1:1 and centrifuged at 7000 RPM for 5 min, and the pellet was spread onto LB agar and incubated at 37 °C for 4–5 h. Cells were harvested from the LB agar and spread onto selective MacConkey agar plates supplemented with kanamycin (32 μg/mL) and nalidixic acid (64 μg/mL). After incubation overnight at 37 °C, 15 transconjugants were screened by PCR for the presence of the IncFIB plasmid.

In addition to generation of IncFIB positive transconjugants, generation of IncFIB plasmid cured strains was attempted by serially passaging strains SE163A, SE417, and SE478 on LB agar plates for up to 34 days. A PCR template was prepared from a single colony at each passage and examined for presence or absence of the plasmid by PCR. Previous studies in our laboratory demonstrated that SE819 is a less virulent isolate lacking several virulence associated plasmids and successfully used as the recipient for conjugation studies [30, 32].

### Growth kinetics

Wild type (SE163A), recipient (SE819), and transconjugant (SE819::IncFIB) strains were grown in LB overnight at 37 °C with shaking (180 RPM). The optical density (OD) was measured at 600 nm, using a Genesys 10UV spectrophotometer (Thermo Electron Corp., Madison, WI). To determine growth kinetics, the overnight cultures of these three strains were inoculated into fresh LB, LB supplemented with ferric chloride (iron-rich), and LB supplemented with 2,2^/^ bipyridyl (iron-depleted) media. The initial OD was adjusted to 0.5 for LB and iron-rich LB. A higher initial OD (3.0) was used in the case of iron-depleted LB to accommodate slow growth under iron deficient conditions. The cell suspensions were incubated with shaking (180 RPM) at 37 °C and the OD was measured in 1 h intervals for 8 h, followed by a final reading at 24 h. Three biological replicates for each sample and each condition were employed and experiments were repeated three times.

### RNA isolation and reverse transcription (RT) PCR

Wild type, recipient, and transconjugant strains were grown in LB, iron-rich, and iron-depleted media supplemented with tetracycline. RNA was isolated from approximately 10^9^ cells collected at mid-logarithmic phase with the Ribopure Bacterial RNA Isolation Kit (Ambion, Invitrogen, Carlsbad, CA), following manufacturer’s instructions. Genomic DNA was removed by treatment with DNase I and the RNA concentration was measured with a NanoDrop spectrometer (Thermo Scientific, Waltham, MA).

Quantitative reverse transcription-PCR (qRT-PCR) was performed to determine expression of the encoded iron acquisition genes on the IncFIB plasmid under iron-rich and iron-depleted growth conditions. cDNA was synthesized from 250 ng RNA using the iScript cDNA synthesis kit (BioRad, Hercules, CA), following manufacturer’s instructions. Primers were designed using the PrimerQuest tool (Integrated DNA Technologies, Coralville, IA) and are listed in Additional file 1: Table S2. The *sitA* primer was designed to detect only transcripts produced from plasmid encoded *sitA* and not from the chromosome. qRT-PCR was performed using iQ SYBR green super-mix and CFX touch real-time PCR detection system (Bio-Rad). The *Salmonella gmk* gene served as endogenous control, to normalize expression [33]. No-reverse-transcriptase (NRT) controls and no-template controls (NTC) were also used to monitor for genomic DNA contamination of the RNA. Differential gene expression and fold differences were calculated by relative quantification (ΔΔ threshold cycle [ΔΔCT]), using Bio-Rad CFX manager software. Two independent experiments using two biological replicates and four technical replicates for each strain were carried out.

### Bacterial invasion assay

Bacterial invasion assays were performed using human intestinal epithelial cells (Caco-2) as described previously [32], with some modifications that were specifically employed for this study. Briefly, 10^5^ Caco-2 cells/well were seeded in 24-well tissue culture plates and incubated at 37 °C overnight. Cells in one of the wells were counted using Cellometer Auto T4 (Nexcelom Bioscience, Lawrence, MA) and the Caco-2 cells were infected with SE819, transconjugant SE819::IncFIB, and wild type SE163A strains at multiplicity of infection (MOI) of 10. After incubation for 1 h at 37 °C, the cells were washed twice with PBS to remove bacteria that had not infected the Caco-2 cells and incubated with 200 μg/ml of gentamicin. After incubation for 1 h at 37 °C, the cells were washed twice with PBS and lysed with 0.1% chilled Triton X-100, followed by dilution and plating on TSA agar to obtain colony forming unit counts (CFUs) of bacteria following overnight incubation at 37 °C. Three replicates per strain were included and the experiments were repeated three times.

To determine influence of iron in invasion of bacteria in to host cells, wild-type (SE163A), recipient (SE819) and transconjugant (SE819::IncFIB) strains were grown overnight in iron-rich and iron-depleted media prior to infection of Caco-2 cells.

### Bacterial persistence assay

Caco-2 cells were infected similar to the invasion assay. After 1 h incubation, the cells were washed twice with PBS and incubated with 100 μg/ml of gentamicin for 48 h. After incubation, cells were washed, lysed, and CFUs were counted as in the invasion assay procedure, with three replicates per strain and experiments carried out in triplicate.

Since variable number of adherence of Caco-2 cells was observed in each experiment, the % invasion and % persistence was determined per 10^6^ Caco-2 cells in order to normalize the number of host cells in different experiments performed. Likewise, number of bacteria for 10 MOI was different for each experiment hence, to normalize this variation between the experiments; % of invasion and % of persistence were determined for each of the strain and plotted the graph as % invasion per 10^6^ Caco-2 cells and % persistence per 10^6^ Caco-2 cells.

### Statistical analysis

Student’s t-test was used to determine statistically significant difference between sample groups, with a *P*-value ≤0.05 considered significant.

## Results

### SNP analysis

**Fig. 1:**
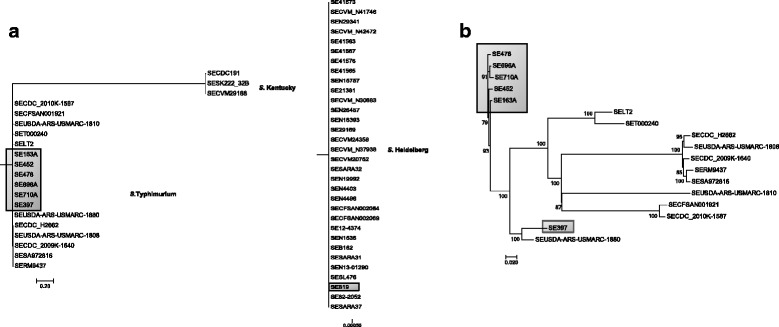
SNP based phylogenetic analysis of *Salmonella enterica* strains isolated from food, animal, and human sources. **a** SNP based evolutionary tree showed that each serovar joined distinct phylogenetic clades, irrespective of presence or absence of an IncFIB plasmid. **b** Five IncFIB containing *S*. Typhimurium isolates (SE163A, SE696A, SE710A, SE452, and SE478) clustered in one clade (shown in the *box*) and SE397 was found to be identical to *S*. Typhimurium USDA-1880. Seven *S*. *enterica* isolates (SE163A, SE696A, SE710A, SE452, SE478, SE397 and SE819) marked with text boxes were sequenced in our study; the genome sequences of the other isolates were obtained from a public data base. Harvest, a core genome SNP mining tool (https://www.cbcb.umd.edu/software/harvest), was used to generate the SNP tree. *S*. *enterica* SL476 was employed as a reference for SNP detection

To understand the role of IncFIB plasmids and how the genes they carry contribute to *Salmonella* virulence, seven *S*. *enterica* strains that were isolated from turkeys and chicken in different geographic locations in the USA were sequenced (Table 1 and Additional file 1: Table S1) [25]. Six of the strains contained IncFIB plasmids and the other (SE819), a less virulent strain, was used as the recipient for conjugation. The isolates containing the IncFIB plasmids were predicted to be serovar Typhimurium using the WGS based *Salmonella* serotyping tool SeqSero, while SE819 was identified as serovar Heidelberg (Table 1). The genome sequences of the isolates were compared to determine evolutionary relatedness to previously sequenced genomes (*n* = 45) of *S*. *enterica* serovars isolated from various food, animal, and human sources (Table 1), using core genome SNP-based phylogenetic analyses. A SNP based evolutionary tree showed each serovar joined distinct phylogenetic clades, irrespective of presence or absence of an IncFIB plasmid (Fig. 1a). To understand the evolutionary relatedness of the *S*. Typhimurium isolates (*n* = 17) including the IncFIB positive strains from the current study, a *S*. Typhimurium-specific phylogenetic tree was constructed (Fig. 1b). It showed five of the IncFIB plasmid containing *S*. *enterica* strains isolated from turkey-associated sources (SE163A, SE696A, SE710A, SE452, SE478) clustered in a single monophyletic clade. The other *S*. *enterica* isolate from chicken (SE397) branched separately together with the *S*. Typhimurium strain (SEUSDA-ARS-USMARC-1880) isolated from a bovine source (Fig. 1b). These data indicate that the five *S*. *enterica* IncFIB plasmid-containing isolates from turkey associated sources have nearly identical chromosomal genetic relatedness.

### Phylogenetic analysis of *sitABCD* and *iucABCD*-*iutA* iron acquisition systems

**Fig. 2:**
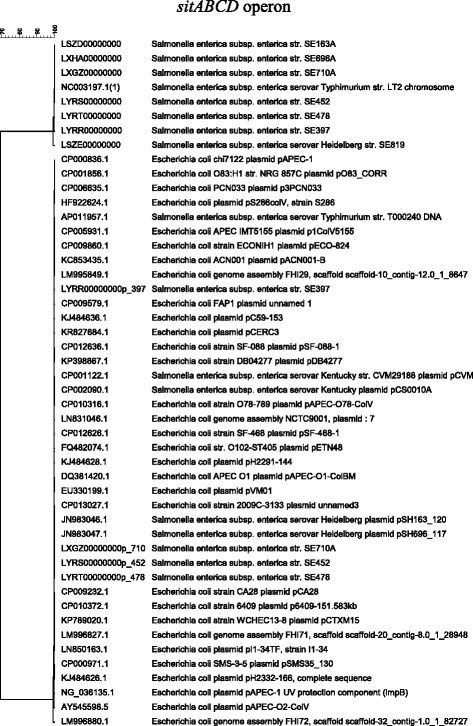
Phylogenetic analysis of the IncFIB plasmid and chromosome encoded *sitABCD* iron transporter. Seven *S*. *enterica* isolates (SE163A, SE696A, SE710A, SE452, SE478, SE397, and SE819) were sequenced in this study and the *sitABCD* sequences of the other isolates were from the NCBI database. All isolates, except for SE819, contained the IncFIB plasmid. The phylogenetic tree was generated using the UPGMA clustering algorithm

**Fig. 3:**
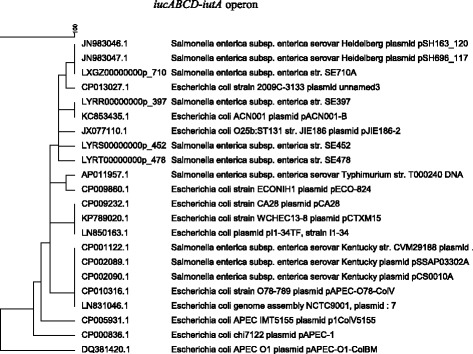
Phylogenetic analysis of the IncFIB plasmid encoded *iucABCD-iutA* iron transporter. Seven *S*. *enterica* isolates (SE163A, SE696A, SE710A, SE452, SE478, SE397, and SE819) were sequenced in this study and the *iucABCD*-*iutA* sequences of the other isolates were from the NCBI database. All isolates, except for SE819, contained the IncFIB plasmid. Since the *iucABCD*-*iutA* transporter is encoded only on a plasmid, the SE819 strain was excluded from analysis. The phylogenetic tree was generated using the UPGMA clustering algorithm

The IncFIB plasmids of *Salmonella* examined in this study encode the Fur regulated iron transporter (*sitABCD*) and aerobactin (*iucABCD-iutA*). The *sitABCD* iron transporter can be encoded both in a plasmid and on the chromosome, whereas the *iucABCD-iutA* iron acquisition system is plasmid-encoded. Interestingly, sequence annotation revealed all six of the IncFIB positive strains possessing the *sit* operon, both in the chromosome and IncFIB plasmid. Phylogenetic analysis of both plasmid and chromosome encoded *sitABCD* transporters from the different *Salmonella* isolates and *E. coli* strains were performed to investigate the genomic diversity of this system (Fig. 2). All chromosome-encoded *sit* operons of *S*. *enterica* clustered in one clade, including the reference LT2 *S*. Typhimurium strain, while the plasmid-encoded *sit* operon of different serovars of *Salmonella* and *E. coli* separated into a distinct clade (Fig. 2). To highlight the differences, PCR primers designed for the plasmid-encoded *sit* genes did not amplify chromosome *sit* genes, as evident by the lack of PCR products for *S*. *enterica* SE819. Our results indicate that plasmid-encoded Sit systems are conserved in both *Salmonella* and *E. coli* while sequence diversity was found between plasmid and chromosome encoded Sit operons in the tested *S*. *enterica* isolates (Fig. 2). The plasmid-encoded aerobactin iron transporter genes were also conserved in the different species of *Salmonella* and *E. coli*, with nearly identical sequences, i.e., ~99% identity (Fig. 3).

### Conjugation and stability of the IncFIB plasmid

To examine the role of the IncFIB plasmid mediated iron acquisition systems of *S*. *enterica*, a transconjugant (SE819::IncFIB) was generated that contained the IncFIB virulence associated plasmid, in which *S*. *enterica* SE819 and SE163A served as recipient and donor, respectively. The presence of the IncFIB plasmid in the transconjugants was confirmed by PCR; transcripts of the IncFIB-associated genes were found in both donor and transconjugant, but not in the recipient strain.

The construction of the transconjugant was essential, since the IncFIB plasmids appear to be stable, in that after 25 to 34 passages SE163A, SE417, and SE478 had not lost the IncFIB plasmid, as evidenced by PCR amplification of the IncFIB replicon. Therefore, addition of the IncFIB plasmid to the less virulent strain was important for evaluating the impact of the plasmid on the virulence of *S*. *enterica*.

### Growth kinetics

**Fig. 4:**
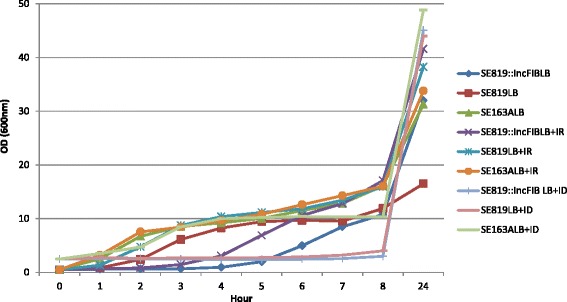
Growth kinetics of *S*. *enterica* strains in iron available in vitro growth media. Wild type (SE163A), recipient (SE819), and transconjugant (SE819::IncFIB) strains were grown in: LB; LB amended with ferric chloride (iron-rich); and LB amended with 2,2^/^ bipyridyl (iron-depleted). Each data point represents the average OD of three replicates. Three independent experiments were performed to examine growth kinetics of the strains. Results of a representative experiment are shown in the graph. ID = iron-depleted and IR = iron-rich

Growth kinetics of donor, recipient, and transconjugant strains were determined by growth in LB, LB supplemented with ferric chloride (iron-rich), and LB supplemented with 2,2^/^ bipyridyl (iron-depleted) (Fig. 4). In general, all grew better in the LB and iron-rich LB than in iron-depleted LB. Highest growth rates of the strains were observed under iron-rich conditions, compared to LB and iron-depleted LB. The donor/wild type *S*. *enterica* SE163A grew better in iron-depleted LB, compared to the recipient and transconjugant strains. Overall, the results showed the three *S*. *enterica* strains demonstrated different growth kinetics, depending on the presence of iron in the growth medium (Fig. 4).

### qRT-PCR

**Fig. 5:**
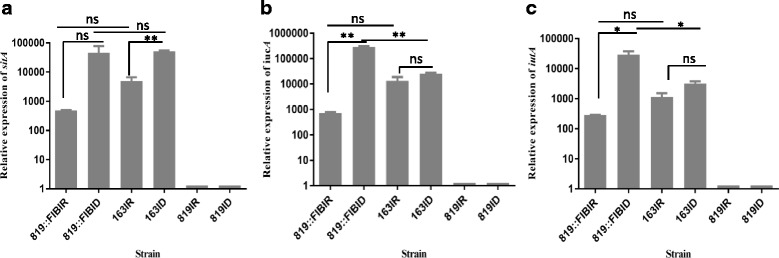
Gene expression of *sitA*, *iucA,* and *iutA* in *S*. *enterica* strains grown under low-iron and high-iron conditions. Differential gene expression was examined in the *S*. *enterica* donor (SE163A), recipient (SE819), and transconjugant (SE819::IncFIB) strains using qRT-PCR and SYBR green assay. **a** relative gene expression of *sitA*; **b** relative gene expression of *iucA*; **c** relative gene expression of *iutA,* shown in a representative experiment. The error bars show the standard error of mean for two biological replicates (consisting of four technical replicates) of each strain. *gmk* was used as a reference gene to normalize the expression of target genes in different samples grown under low and high iron conditions. SE819 strain which does not possess IncFIB plasmid used as control to normalize the background gene expressions. Student’s t-test was performed to determine the statistically significant difference between two groups of samples. A *p* value ≤0.05 was considered a significant difference between the two groups compared, as indicated by the asterisks (ns = not significant)

To determine gene expression of plasmid encoded Sit and aerobactin operons, the transcripts of the *sitA*, *iucA*, and *iutA* genes in wild type, transconjugant, and recipient strains grown in iron-rich and iron-depleted LB were examined using qRT-PCR*.* Increased level (~3 log) of *sitA* expression was found in the transconjugant strain grown under iron-depleted compared to iron-rich conditions (Fig. 5a). However, due to variation among replicates, the difference was not statistically significant. A significantly increased level of *sitA* expression was observed in SE163A in the iron-depleted growth medium, as compared with iron-rich (Fig. 5a). As expected, the *sitA* transcript was not detected in SE819, which lacked the plasmid. The expression of *iucA* in the transconjugant strain in iron-depleted conditions was significantly greater (~3 log), compared to iron-rich (Fig. 5b), while expression was similar for SE163A in both the iron-depleted and iron-rich media (Fig. 5b). Results of *iutA* gene expression were similar to *iucA,* with a ~ 2.5-fold difference of expression for transconjugants and similar levels of expression for *S*. *enterica* SE163A (Fig. 5c).

### Invasion and persistence in Caco-2 cells

**Fig. 6:**
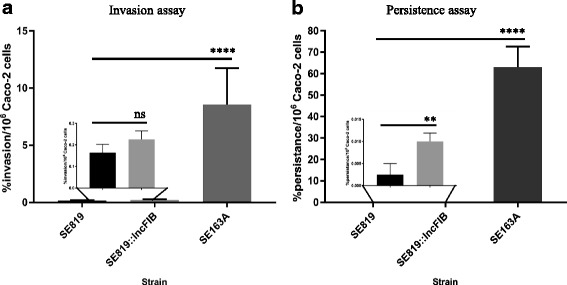
Invasion and persistence assays of different strains of *S*. *enterica*. Human intestinal epithelial cells (Caco-2) were infected with recipient SE819, transconjugant and wild type SE163A of *S*. *enterica* at MOI of 10. Bacterial invasion (**a**) and persistence assays (**b**) were performed 1 h and 48 h post infection, respectively. A slightly higher uptake and increased persistence was observed when Caco-2 cells were infected with the transconjugant strain as compared to the recipient SE819 strain. The error bars show the standard error of means for three biological replicates of three independent experiments. Student’s t-test was performed to determine the statistically significant difference between two groups of sample. A *p* value ≤0.05 was considered a significant difference between the two groups compared, as indicated by the asterisks (wild type vs. recipient and transconjugant vs. recipient). Statistically non-significant differences are indicated by “ns”

To assess the role of the IncFIB plasmid in invasion and persistence in host cells, human intestinal epithelial cells (Caco-2) were infected with recipient, transconjugant, and wild type strains of *S*. *enterica*. Significantly increased invasion and persistence of the wild type in Caco-2 cells was observed, compared to transconjugant and recipient strains. A slightly higher uptake was observed in the transconjugant, compared to the recipient (Fig. 6a). However, this difference was not statistically significant by Student’s t-test. A significantly higher number of transconjugants were found to persist in Caco-2 cells, compared to the recipient strain (Fig. 6b).

**Fig. 7:**
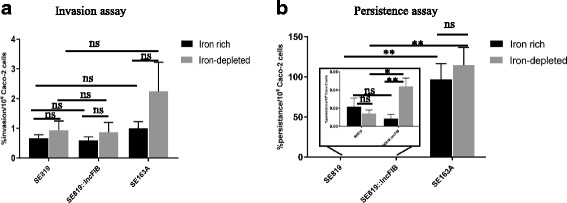
Invasion and persistence assays of different strains of *S*. *enterica* grown in iron-rich and iron-depleted conditions prior to infection. Recipient SE819, transconjugant and wild type SE163A of *S*. *enterica* strains were grown in iron-rich and iron-depleted media overnight prior to infect human intestinal epithelial cells (Caco-2) with at MOI of 10. Bacterial invasion (**a**) and persistence assays (**b**) were performed 1 h and 48 h post infection, respectively. The error bars show the standard error of means for 2–3 biological replicates of two independent experiments. Student’s t-test was performed to determine the statistically significant difference between two groups of sample. A *p* value ≤0.05 was considered a significant difference between the two groups compared, as indicated by the asterisks. Statistically non-significant differences are indicated by “ns”

Both recipient and transconjugant strains invaded in Caco-2 cells at similar rates when grown in iron-rich and iron-depleted media prior to infection (Fig. 7a). Wild type strain invaded at higher rate in both iron-rich and iron-depleted conditions than the recipient and transconjugant, however, this difference was not statistically significant (Fig. 7a). When grown in iron-rich media, the transconjugant persisted at lower rate in Caco-2 cells as compared the recipient; however, this difference was not statistically significant. Whereas, in iron-depleted media, the transconjugant persisted significantly higher rate than the recipient (Fig. 7b). Interestingly, transconjugant persisted at higher rate when grown in the iron-depleted than the iron-rich media (Fig. 7b). Wild type strain persisted significantly higher rate in both iron-rich and iron-depleted conditions than the recipient and transconjugant. Unlike transconjugant, wild type strain showed similar persistence rate both in iron-rich and iron-depleted conditions (Fig. 7b).

## Discussion

Dissemination of plasmid mediated antimicrobial resistance and virulence genes of bacterial pathogens is a persistent health concern [6, 34]. A better understanding of the contribution of specific types of plasmids to antimicrobial resistance and virulence is important for development of novel strategies to control the spread of these plasmids among foodborne pathogens. In the present study, the genetic background of *S*. *enterica* isolates containing IncFIB plasmids and the iron acquisition systems encoded by these plasmids were analyzed by both in silico analysis and laboratory based experiments.

SNP analysis of genomes of a number of *S*. *enterica* strains representing serovars Heidelberg, Typhimurium, and Kentucky isolated from food, animal, and human sources, revealed serovar specific clades (Fig. 1a), indicating paraphyletic relationships. More importantly, within the *S*. Typhimurium clade, five of the IncFIB plasmid containing turkey-associated isolates formed into a unique single monophyletic subclade distinct from the other sequences analyzed (Fig. 1b). It is interesting to note that the IncFIB plasmid was integrated into the chromosome of *S.* Typhimurium SET000240 after isolation from a human patient, indicating IncFIB encoded genes have the potential to be maintained as a plasmid with the ability to integrate into the chromosome [35]. Hence, this plasmid has the potential to disseminate by horizontal gene transfer into *Salmonella* and other pathogens.

Previous studies have shown that IncFIB plasmids have the potential to contribute to increased colonization and fitness in the chick embryo and/or other avian animal models when infected with avian pathogenic *E. coli* [36, 37] and *S*. Kentucky [10]. Hence, dissemination of the IncFIB plasmid into *Salmonella* serovars, such as Typhimurium and Heidelberg, which are known to cause human infections, may contribute to improved survival in food animals, resulting in increased opportunities for human infection via contaminated food products. In addition to encoding factors contributing to virulence, IncFIB plasmids in *Salmonella* often carry multiple antimicrobial resistance genes [6], which is problematic if they cause diseases requiring antimicrobial therapy. The identification and evaluation of specific factors relative to colonization, virulence, and plasmid dissemination are important for finding effective ways to control these foodborne pathogens and reduce spread of virulence and antimicrobial resistance plasmids in the food production environment and exposure to humans.

Iron is one of the key signaling elements that helps pathogens sense their environment as well as regulate gene expression to survive under reduced iron conditions in human and animal hosts [13, 38, 39]. Bacterial pathogens are known to possess iron transporter systems that facilitate survival under low iron conditions [9, 21, 22]. In the present study, the focus was on plasmid-encoded iron transporter systems encoded by *sitABCD* and *iucABCD*-*iutA*. *sitABCD* genes are present both in the chromosome and on the IncFIB plasmids of the six isolates whose genomes have been sequenced in this study; however, there appears to be significant sequence diversity between the two *sitABCD* operons (Fig. 2). Degree of virulence depending on whether the *sitABCD* transporters are encoded on both the chromosome and the plasmid versus one or the other may influence the virulence of pathogen. It is known that chromosome encoded *sitABCD* play role in virulence [15], however, currently there is scarcity of study evaluating the role of plasmid encoded *sitABCD* in virulence of *S*. *enterica* and other pathogens. Also, it remains to be determined whether presence of both *sitABCD* will contribute in virulence as synergistic manner. We speculate that plasmid and chromosomally encoded *sitABCD* transporters may exhibit differential expression within the host and the natural environment. Further research is needed to substantiate this conclusion.

The IncFIB plasmids demonstrated stability in in vitro culture, as evidenced by retention of the plasmid after repeated passaging. Host addiction genes (*vagCD* and *ccdAB*), encoded on this plasmid likely contribute to stability of the plasmid [40–42] and is indicative of the plasmid’s importance to the bacterial cell in food animal environments. While stably maintained in the bacterial host, copies of the plasmid are able to be transferred to recipients. The IncFIB plasmid was able to be transferred from *Salmonella* to *E. coli* and vice versa via conjugation, confirming the IncFIB to be a conjugative plasmid.

During the growth kinetics experiments, wild type, recipient, and transconjugant strains all showed slower and/or reduced growth under low iron conditions when compared to high iron conditions (Fig. 4), indicating the importance of iron for replication and growth of these *Salmonella* isolates and supporting results of previous studies [10, 43]. In addition, SE819 (recipient) and SE819::IncFIB (transconjugant) showed similar growth kinetics in vitro in iron-depleted media. The reason why transconjugant did not show any growth advantage in iron-depleted growth media is currently unknown. Since, wild type strain (SE163A) showed increased growth kinetics in iron-depleted media compared to the recipient and transconjugants; therefore additional factors may require in addition to IncFIB plasmid to obtain growth advantages in iron-depleted condition. Additional experiments are essential to test this hypothesis.

Expression of the plasmid encoded *sitA*, *iucA*, and *iutA* genes were greater under iron-depleted conditions compared with iron-rich conditions in the transconjugant SE819::IncFIB, while increased *sitA* transcript was observed only under iron-depleted conditions in the case of SE163A. *iucA* and *iutA* of SE163A were expressed similarly under both iron-rich and iron-depleted growth media. The cause of the observed difference in expression profiles of wild type and the transconjugant remains unknown, but is likely related to differences in the bacterial genomes or other plasmids present in the wild type strain. Botteldoom et al. [33] investigated the expressions of the different housekeeping genes (16S rRNA, *rpo* *D* and *gmk*) of *Salmonella enterica* in different growth conditions by qRT-PCR. They demonstrated that expression of *gmk* was more stable than the other two housekeeping genes in various growth conditions tested. In our initial experiment, we examined both 16S rRNA and *gmk* as reference genes for normalizations; we also observed that expression of *gmk* was stable than 16S rRNA in different growth conditions. Therefore, in the further experiments, we used *gmk* as the reference gene to normalize the gene expressions in qRT-PCR.

Fur is an iron regulatory component and is a well-studied transcription repressor of target gene(s) repression in response to iron [44–46]. Fur binds to consensus regions upstream of target genes Fur-box [47, 48]. A previous study identified consensus sequences of Fur-box in the *S.* Typhimurium genome [48] and several studies have suggested that Fur regulates directly or indirectly the many virulence factors of pathogens, including shiga-like toxins in *E. coli* [47], colonization capacity of *Helicobacter pylori* in the human stomach [13], biofilm formation of *Vibrio* species, and Type 3 secretion systems of *Shigella* and *S.* Typhimurium [11]. The chromosome encoded *sitABCD* in *S.* Typhimurium is regulated by Fur, an iron transporter required for virulence [15]. The mechanisms of regulation of plasmid-encoded genes of *sitABCD* and *iucABCD-iutA* operons in response to iron have yet to be determined.

In the invasion assays, a slightly higher percentage uptake of the transconjugant strain was observed compared to the recipient (Fig. 6a). Similarly, the percentage of persistence in the host was significantly higher in the transconjugant than recipient (Fig. 6b), indicating IncFIB plasmid contributes to invasion and persistence. However, the specific mechanisms are not known. Additionally, the wild type strain SE163A showed significantly higher invasion and persistence in Caco-2 cells compared to transconjugant and recipient (Fig. 6a & b). In addition, transconjugant persisted at higher rate when grown in iron-depleted than the iron-rich media prior to infection while, wild type strain showed similar persistence rate in both iron-rich and iron-depleted conditions (Fig. 7b). The distinct persistence of wild type and tansconjugant when grown in iron-rich and iron-depleted conditions prior to infection of Caco-2 cells may be due to the difference in the genetic backbone of these strains. From these observations we speculate that factors encoded on the IncFIB plasmid contribute synergistically to facilitate uptake and persistence in the host. Whereas additional virulence factors, which are either encoded by other virulence associated plasmid(s) or reside on the chromosome, contribute to increased infectivity and fitness within host cells. However, we have to interpret of these findings cautiously as wild type strain possesses several virulence associated plasmids which is absent in the recipient strain. Additionally, substantial nucleotides sequence diversity were found in the different virulence associated genes examined such as *phoQ*, *sdiA*, *sinH*, *avrA*, *ssaQ*, *sopB*, *bcfC*, *htpG*, *iroN*, *eutR*, *ssaC*, *sifA*, and *spaN* encoded on the chromosome between SE819 (recipient) and other six isolates including wild type strain SE163A (data not shown). Future studies would be helpful to examine the role of plasmid encoded factors associated with IncX4, IncA/C, and/or IncI1 plasmids often co-located with IncFIB plasmids, in both invasion and the persistence of host cells, by generating transconjugants containing combinations of plasmids.

## Conclusions

In conclusion, it has been shown that there is minimal sequence diversity in the iron acquisition systems of IncFIB plasmids present among different bacterial species, including foodborne *Salmonella* serovars*,* suggesting that similar plasmid encoded virulence factor(s) may disseminate among bacterial pathogens. The results of the study show that the IncFIB plasmids contribute to an increased ability to persist in intestinal epithelial cells as well as the virulence potential of an organism. Thus, because IncFIB plasmids have the potential to carry both virulence and antimicrobial resistance genes, they represent a health concern since transfer of a single plasmid to a susceptible bacterial strain can render it both more virulent and resistant to multiple antimicrobial agents.

## Additional file

Additional file 1: Table S1.
*Salmonella* spp. that were sequenced and constructed for this research. **Table S2.** Primers employed for qRT-PCR (PDF 66 kb)

## Abbreviations

IncFIB: Incompatibility group (Inc) FIB plasmid
*S. enterica*: *Salmonella enterica*
SNP: Single nucleotide polymorphism
WGS: Whole genome sequencing
