# Supplementary material for: Comparative genomic analysis and characterization of incompatibility group FIB plasmid encoded virulence factors of Salmonella enterica isolated from food sources
Source: BMC Genomics. 2017 Aug 2;18:570. doi: 10.1186/s12864-017-3954-5 (PMC5541697; doi:10.1186/s12864-017-3954-5)
Supplement: Additional file 1: Table S1. — Salmonella spp. that were sequenced and constructed for this research. Table S2. Primers employed for qRT-PCR (PDF 66 kb) [file 12864_2017_3954_MOESM1_ESM.pdf]

Table S1. *Salmonella* spp. that were sequenced and constructed for this research

Strain No.	Strain	Application in the study
SE163A	*S*. Typhimurium[c]	WGS[a] for SNP[b] analysis (wild type strain)
SE819	*S*. Heidelberg[c]	WGS for SNP analysis (recipient strain)
SE696A	*S*. Typhimurium[c]	WGS for SNP analysis
SE710A	*S*. Typhimurium[c]	WGS for SNP analysis
SE397	*S*. Typhimurium[c]	WGS for SNP analysis
SE452	*S*. Typhimurium[c]	WGS for SNP analysis
SE478	*S*. Typhimurium[c]	WGS for SNP analysis
J53	*E. coli*	Conjugation (recipient)
J53::IncFIB	Transconjugant	Conjugation (J53 strain containing IncFIB plasmid from donor SE163A strain)
SE819::IncFIB	Transconjugant	Conjugation (SE819 strain containing IncFIB plasmid from donor SE163A strain)

[a] WGS; Whole Genome Sequence
[b] SNP; Single Nucleotide Polymorphism
[c] *Salmonella* serotypes were predicted using SeqSero (www.denglab.info/SeqSero)

Table S2. Primers employed for qRT-PCR

Gene	Primer sequence (5′ to 3′)	Amplicon
sitA	*sitA*F: CTACCAACGTAATGCCGATACT	104 bp
	*sitA*R: TGACCATCCATCGCTGATTC	
iucA	*iucA*F: TTACGGCTGAAGCGGATTAC	108 bp
	*iucA*R: CCTGGCAGTCACGGTAAATAA	
iutA	*iutA*F: AGCGTGGTGGCGAATAAA	114 bp
	*iutA*R:TCCGGTACTCCAGTCAGTATC	
gmk	*gmk*F: TTGGCAGGGAGGCGTTT	62 bp
	*gmk*R: GCGCGAAGTGCCGTAGTAAT	
